# Sleep Quality in Adolescents and Adults With Cystic Fibrosis Taking Elexacaftor/Tezacaftor/Ivacaftor

**DOI:** 10.1016/j.chpulm.2026.100280

**Published:** 2026-07-09

**Authors:** Melissa H. Ross, Christine R. Ford, Kristin A. Riekert, Robin S. Everhart, Emily F. Muther, Christina L. Duncan, Jordana E. Hoppe, Megan von Isenburg, Emma Lyons, Michelle Prickett

**Affiliations:** a Northwestern University School of Medicine, Chicago, IL; b Johns Hopkins University, Baltimore, MD; c Virginia Commonwealth University, Richmond, VA; d University of Colorado Anschutz Medical Campus, Aurora, CO; e Oklahoma State University, Stillwater, OK; f Community Partner, Durham, NC

To the Editor:

Highly effective Cystic Fibrosis Transmembrane Conductance Regulator (CFTR) modulator therapies (HEMTs), including elexacaftor/tezacaftor/ivacaftor (ETI), have dramatically increased the predicted life expectancy of someone born with cystic fibrosis (CF) to 65 years of age.^1^ With most people with CF (PwCF) taking HEMT living well into adulthood, there is an urgent need to learn more about the impacts of CF and HEMT on various domains of aging and health-related quality of life, including the impact on sleep. PwCF not taking HEMT experience poor sleep, with likely underlying etiologies including cough, frequent stooling, reflux, chronic pain, and hypoxia.^2^ PwCF may also have circadian rhythm disturbances because of defective hypothalamic CFTR function.^3^ Severity of lung disease is correlated with severity of sleep impairments, with the worst sleep reported by those awaiting lung transplant.^4^^,^^5^

Researchers expected improvements in sleep to follow improvements in pulmonary symptoms for PwCF taking ETI. However, this has not been consistently observed. A few small studies have examined sleep when considering mental health side effects of ETI, finding mixed results. Although some studies show stability or improvement in subjective sleep quality, many show worsening of self-reported sleep quality after ETI initiation.^6^, ^7^, ^8^, ^9^, ^10^ Given the far-reaching health consequences of poor sleep, including mental health disorders, obesity, insulin resistance, and increased susceptibility to infection, it is vital to learn more about sleep in PwCF taking ETI.

## Methods

The Your ETI (yETI) study was a cross-sectional, multicenter study conducted from March to June 2024 at 18 US CF care centers in the CF Foundation’s Success with Therapies Research Consortium. This study obtained ethics approval from the Boston Children’s Hospital institutional review board (No. IRB-P00046466). Eligible participants were English speaking PwCF ≥ 7 years of age who had been prescribed ETI for at least 18 months. Exclusion criteria included transplant listing and pregnancy. Participants completed a survey including validated measures of health and well-being and demographic surveys (completed by caregivers for PwCF < 13 years of age). Clinical characteristics were abstracted from the medical record, including BMI (BMI or BMI percentile for < 20 years of age) and lung function (best % predicted FEV_1_ in the past year). Analyses were restricted to participants ≥ 13 years of age who completed the sleep quality measure.

### Statistical Analysis

Sleep quality was measured using the Pittsburgh Sleep Quality Index (PSQI), with poor sleep defined as a total score > 5. Depressive and anxiety symptoms were screened with the 8-item Patient Health Questionnaire and the Generalized Anxiety Disorder 7-item scale, respectively, with scores ≥ 10 considered a positive screen.

Participant characteristics were summarized descriptively, and prevalence ratios (PRs) with 95% CIs for poor sleep, as previously defined, were estimated using Poisson regression with robust SEs. Both unadjusted models and models adjusted for age group and sex were conducted. Missing data were handled using listwise deletion. Analyses were conducted in SAS 9.4 (SAS Institute).

## Results

A total of 300 participants were included in the analyses. The mean age ± SD was 28.3 ± 12.51 years (range, 13-74 years). Most were female, White non-Hispanic, and working or in school full time. Approximately one-third were overweight or obese, nearly one-quarter had FEV_1_ % predicted < 70%, and 17% screened positive for depression or anxiety.

The mean PSQI score ± SD was 6.2 ± 3.9, and 60% met the criteria for poor sleep quality (Table 1). Although 55% rated their overall sleep quality as fairly good, 20% reported fairly bad or very bad sleep quality. The sleep disturbances component, which sums the frequency of common nighttime problems including nocturnal awakenings, bathroom use, or discomfort, indicated that 28% experienced ≥ 10 nightly disturbances, reflecting moderate to severe disruption. Of the patients, 16% reported using sleep medications ≥ 3 times per week. Daytime dysfunction was minimal for most, with 45% scoring 0, indicating no dysfunction.

**Table 1:** Participant Sleep Quality (N = 300)

Variable	Value
Total score (PSQI)	6.21 [3.89]
Poor sleep quality	180 (60.0)
Subjective sleep quality^a^	
Very good	73 (24.3)
Fairly good	165 (55.0)
Fairly bad	55 (18.3)
Very bad	7 (2.3)
Sleep latency, min^b^	
< 15	78 (26.0)
16-30	113 (37.7)
31-60	61 (20.3)
> 60	48 (16.0)
Sleep duration, h^c^	
> 7	144 (48.0)
6-7	77 (25.7)
5-6	74 (24.7)
< 5	5 (1.7)
Habitual sleep efficiency, %^d^	
> 85	190 (63.3)
75-84	69 (23.0)
65-74	19 (6.3)
< 65	22 (7.3)
Sleep disturbances^e^	
0	17 (5.7)
1-9	199 (66.3)
10-18	73 (24.3)
19-27	11 (3.7)
Sleep medication use^f^	
Not during the past month	219 (73.0)
Less than once a week	22 (7.3)
Once or twice a week	12 (4.0)
≥ 3 times a week	47 (15.7)
Daytime dysfunction^g^	
0	134 (44.7)
1-2	127 (42.3)
3-4	33 (11.0)
5-6	6 (2.0)

Data are presented as mean [SD] or No. (%). Percentages may not add to 100% because of rounding. PSQI = Pittsburgh Sleep Quality Index. The PSQI global score ranges from 0 to 21 with higher scores signifying poorer sleep quality; a score > 5 suggests significant sleep difficulties or poor sleep quality. Component scores are scored from 0 to 3, with higher scores denoting greater impairment.

a Indicates perceived overall sleep quality.

b Indicates the time to fall asleep.

c Indicates total hours of sleep.

d Indicates the ratio of hours slept to hours in bed.

e Indicates the frequency of nighttime disruptions.

f Indicates the frequency of prescribed or over-the-counter sleep aids.

g Indicates daytime sleepiness and impaired functioning.

Poor sleep was more prevalent in adults than adolescents 13 to 17 years of age, with PRs of 1.86 (95% CI, 1.34-2.57) for patients 18 to 25 years of age, 1.54 (95% CI, 1.07-2.21) for patients 26 to 35 years of age, and 1.83 (95% CI, 1.32-2.55) for patients ≥ 36 years of age (Table 2). There were no statistically significant differences in poor sleep by sex, weight status, or work/school status (Table 2). Poor sleep was more likely among participants with FEV_1_ < 70% (PR, 1.44; 95% CI, 1.21-1.71) and those screening positive for depression or anxiety (PR, 1.72; 95% CI, 1.49-1.98). Adjustment for age group and sex did not change the direction or statistical significance of any findings (Table 2).

**Table 2:** Sleep Scores, Prevalence of Poor Sleep, and Prevalence Ratios (N = 300)

Variable	No.	PSQI Total Score	Prevalence of Poor Sleep	Unadjusted Prevalence Ratio (95% CI)	Adjusted Prevalence Ratio (95% CI)
Overall	300	6.21 [3.89]	180 (60.0)	NA	NA
Age group, y					
13-17 (ref)	71	4.25 [2.84]	27 (38.0)	NA	NA
18-25	85	6.60 [3.74]	60 (70.6)	**1.86 (1.34-2.58)**	**1.84 (1.33-2.55)**
26-35	65	6.42 [4.03]	38 (58.5)	**1.54 (1.07-2.21)**	**1.51 (1.05-2.17)**
≥ 36	79	7.39 [4.16]	55 (69.6)	**1.83 (1.32-2.55)**	**1.81 (1.30-2.52)**
Sex assigned at birth					
Male (ref)	130	5.49 [3.44]	70 (53.9)	NA	NA
Female	170	6.76 [4.12]	110 (64.7)	1.20 (0.99-1.46)	1.18 (0.97-1.42)
Work or school status					
Not working or in school full time (ref)	91	6.95 [4.11]	60 (65.9)	NA	NA
Working or in school full time	209	5.89 [3.76]	120 (57.4)	0.87 (0.72-1.05)	1.00 (0.83-1.20)
BMI category					
Not overweight or obese (ref)	203	5.97 [3.68]	116 (57.1)	NA	NA
Overweight or obese	97	6.72 [4.27]	64 (66.0)	1.15 (0.96-1.39)	1.13 (0.95-1.35)
Lung function					
FEV_1_ % predicted ≥ 70% (ref)	245	5.83 [3.76]	136 (55.5)	NA	NA
FEV_1_ % predicted < 70%	55	7.91 [4.04]	44 (80.0)	**1.44 (1.21-1.71)**	**1.33 (1.10-1.60)**
Screened positive for depression or anxiety					
No (ref)	250	5.50 [3.56]	134 (53.6)	NA	NA
Yes	50	9.80 [3.50]	46 (92.0)	**1.72 (1.49-1.98)**	**1.67 (1.45-1.93)**

Data are presented as No. (%), mean [SD], or as otherwise indicated. Prevalence ratios and 95% CIs were estimated using unadjusted Poisson regression with robust SEs. Reference groups are indicated for each variable. Adjusted models include age group and sex. Models for age group are adjusted for sex, and models for sex are adjusted for age group. Bolded values indicate significance at α = 0.05. PSQI = Pittsburgh Sleep Quality Index. Poor sleep is defined as a PSQI score > 5. NA = not applicable; ref = reference.

## Discussion

To our knowledge, this is the largest study evaluating sleep quality in PwCF taking ETI. In this study population, 60% of participants had poor sleep quality based on PSQI score; however, only 20% of respondents self-rated their sleep as poor. This suggests that sleep remains a significant challenge for PwCF taking ETI despite improvements in many of the symptoms once thought to be the drivers of sleep disturbance in PwCF. Encouragingly, the percentage of participants who perceived their sleep as poor is lower than traditionally has been reported in pre-HEMT studies^2^^,^^5^; importantly, only 2% of participants experienced severe daytime dysfunction. The disconnect between elevated PSQI scores and most participants rating their sleep as fairly good may reflect that PwCF have habituated to poor sleep or is relative to their sleep before starting ETI. Alternatively, mild sleep disturbances across multiple different components can result in an elevated PSQI score. This finding suggests that querying about individual sleep components may help providers better identify and address sleep problems.

In this study, participants with lower lung function (FEV_1_ % predicted < 70%) remained more likely to experience poor sleep than participants with preserved lung function.^4^^,^^5^ Adults were more likely to experience poor sleep than adolescents, as were participants with comorbid depression or anxiety.

Strengths of this study include its large sample size of both adolescents and adults drawn from 18 different CF centers around the country. An important limitation of this study is its inability to detect changes in sleep quality after ETI initiation because of its cross-sectional design. Additionally, the study inclusion criteria of 18 months of stability on ETI may have excluded patients who stopped the medication because of sleep-related side effects. This study illustrates that sleep remains a significant challenge for PwCF on ETI therapy and highlights the need for clinical monitoring of sleep for PwCF taking ETI and future research into its causes.

## Funding/Support

This work was supported by the Cystic Fibrosis Foundation [Grants YETI23PE0, RIEKER23PE0, SAWICK14PE1].

## Financial/Nonfinancial Disclosures

The authors have reported to *CHEST Pulmonary* the following: C. F., R. S. E., E. F. M., M. v. I., and E. L. have received support from the Cystic Fibrosis Foundation. K. A. R. has received support from the Cystic Fibrosis Foundation and the NIH (NIDDK, NHLBI). C. L. D. has received support from the Cystic Fibrosis Foundation and is on the Board of Directors of the Society of Pediatric Psychology. J. E. H. has received support from the Cystic Fibrosis Foundation, Cystic Fibrosis Canada, and the NIH; and has consulted for Vertex Pharmaceuticals. M. P. has received support from the Cystic Fibrosis Foundation and Becton Dickinson. None declared (M. H. R.).
